# Prevalence of Intestinal Parasite Infections and Associated Factors among Pregnant Women in Northwest Ethiopia

**DOI:** 10.1155/2022/9065425

**Published:** 2022-05-09

**Authors:** Yibeltal Aschale, Awoke Minwuyelet, Tadesse Yirga Akalu, Asmare Talie

**Affiliations:** ^1^Department of Medical Laboratory Science, College of Health Science, Debre Markos University, Debre Markos, Ethiopia; ^2^Medical Parasitologist, Bichena Primary Hospital, Amhara Region, Ethiopia; ^3^Department of Pediatrics and Child Health, College of Health Science, Debre Markos University, Debre Markos, Ethiopia; ^4^Department of Midwifery, College of Health Science, Debre Markos University, Debre Markos, Ethiopia

## Abstract

**Background:**

Intestinal parasite infections are among the most common infections worldwide. They pose a high burden in pregnant women in developing countries causing maternal anemia, low birth weight, and prenatal mortality. This study is aimed at assessing intestinal parasite infection prevalence, species diversity, and associated factors among pregnant women.

**Methods:**

A community-based cross-sectional study was conducted among pregnant women in Debre Elias district from March 2021 to July 2021. Three hundred sixty-three study participants meeting the inclusion criteria were enrolled for the study, and all submitted the required amount and quality of stool specimen. Immediately after collection, macroscopic (gross) and microscopic (saline wet mount) examination of stool sample was performed to detect and identify intestinal parasites. The generated data were checked for completeness, coded, entered, and analyzed using SPSS version 20.0 (SPSS Inc., Chicago, 2011) software. Binary logistic regression was applied to show significant association between dependent and independent variables. Statistically significant association was declared at a *P* value of < 0.05.

**Result:**

Of the study participants screened for intestinal parasite, 43.5% (158/363) were infected with at least one intestinal parasite. From the total positives, 40.5% (147/363) were mono and 3.0% (11/363) were double infections. Five intestinal parasite species were recorded, of which hookworm was the predominant, (65.2%, 103/158) followed by *E. histolytica/dispar* (12.7%, 20/158) and *G. lamblia* (11.4%, 18/158). *Ascaris lumbricoides* and *Taenia species* comprised the least percentage (1.9%, 3/158 each). Source of drinking water and occupation were identified as significant factors associated with intestinal parasite infection. Farmer pregnant women were 6.41 times (AOR = 6.41, 95% CI: 1.05-39.16; *P* = 0.034) more likely to be infected by intestinal parasites than their counterparts. Pregnant women who drank tape water were 0.52 times less (AOR = 0.52, 95% CI: 0.30-0.88; *P* = 0.017) likely to be infected by intestinal parasites.

**Conclusion:**

Intestinal parasite infections remained a serious health burden to pregnant women in the study area with the dominance of a hematophagous worm (hookworm). Community-based intestinal parasite screening and treatment are essential to alleviate the burden caused by intestinal parasite infections.

## 1. Background

Intestinal parasitic infections caused by pathogenic helminth and protozoan species are prevalent in the world, mainly in developing countries [1]. They are highly prevalent among the poor sections of the population due to poverty, poor living condition, poor waste disposal systems, and lack of safe water supply [2, 3]. Epidemiological evidence suggested that an estimated two billion people in the world were infected with intestinal parasites caused by helminth and protozoa [4]. These infections are responsible for iron deficiency anemia, vitamin deficiency, and severe chronic diarrhea [5, 6]. In sub-Saharan African countries, 200-250 million people are infected with at least one or more species of intestinal nematodes [7].

According to WHO 2006 report, schoolchildren, women aged 15-49, and pregnant women are more susceptible to IPIs [8]. Intestinal parasites during pregnancy, if left untreated, cause a great burden on the mother, growing fetus, and newborn. They can lead to maternal anemia, nutritional deficiencies, immune suppression, and organ blockages [9]. Globally, hookworm, *A. lumbricoides*, and *T. trichuria* are the three commonest helminth infections, and *E. histolytica/dispar* and *G. lamblia* are common protozoan infections among pregnant women [10, 11].

The degree of severity of intestinal parasite infections depends on parasite factors (parasite strain, parasite load, and specific tissue/organ affected), host factors (age, nutritional and immunological status, general health, and coexisting disease), and socioeconomic factors. Therefore, it is difficult to evaluate the burden caused by intestinal parasitic infections as so many infections are asymptomatic and therefore remain undetected [2]. The most important challenge of IPI is that about 90% of infected individuals remain asymptomatic at diagnosis [12].

Intestinal parasite magnitude in Ethiopia is high due to favorable temperature and humidity, environmental and sociocultural practices, and poverty [13]. There was a high prevalence of helminthic infection in the lower altitudes, and *Ascaris lumbricoides* is the most predominant intestinal parasite in different localities occurring together with *Trichuris* and hookworm [14–16]. Although several studies have been carried out and intervention methods are applied to control and prevent IPI in Ethiopia, information on the prevalence and distribution of intestinal parasites among pregnant women is inadequate and not updated periodically. This study is aimed at assessing intestinal parasite infection prevalence and associated factors among pregnant women.

## 2. Material and Methods

### 2.1. Study Area

The study was conducted among pregnant women in the Debre Elias district, Northwest Ethiopia, which is located approximately 42 km southwest of the East Gojjam Zone capital city, Debre Markos, 248 km far from Bahir Dar, capital of Amhara regional state, and 340 km far from Addis Ababa. It has a total of 17 kebeles of which 16 are rural and one is urban. The district is the source of wheat which is the main source of food and industrial input. The district is divided into two major agroecological zones. The moist midaltitude (Woynadega) comprises 91% of the district (2100-2300 meters above sea level), and the warm moist land (Kolla) comprises 9% of the Woreda (1500-2100 meters above sea level). The average annual temperature is 14.9°C. There are four health centers rendering services for inhabitants of the district.

### 2.2. Study Design and Period

A community-based cross-sectional study was conducted among pregnant women from March 2021 to July 2021 to assess the prevalence, species diversity, and associated factors of intestinal parasite infection.

### 2.3. Study Population Characteristics and Sampling Techniques

Three kebeles (the smallest administrative unit in Ethiopia) were selected using the lottery method from the district. Determination and allocation of the proportional sample size for each selected kebele were made based on the updated number of pregnant women found at the health extension worker's registry in each kebele health post. The required sample size was estimated by using single population proportion formula taking intestinal parasite prevalence of 31.3% [17] assuming a 95% confidence level, 5% margin of error. Finally, 363 study participants meeting the inclusion criteria were selected using systematic random sampling. Participants who were conscious, able to give a stool sample, and those not on antiparasitic medication were included, and those with severe medical conditions were excluded.

### 2.4. Data Collection and Laboratory Methods

#### 2.4.1. Sociodemographic Data Collection

A semistructured questionnaire that addresses all sociodemographic data and the risk factors around was developed in the English version. It was then translated into the local language of the area (Amharic). Finally, the interview was conducted by trained data collectors.

#### 2.4.2. Stool Sample Collection, Processing, and Examination

A single stool specimen of about 5-7 g was collected into a dry, clean, and leak-proof plastic stool-cup (30 ml capacity) container after appropriate instruction. Stool samples were transported to the nearby health center laboratory. Gross examination (quantity, color, consistency, appearance, and presence of intact worm and/or tapeworm segment) was performed before the microscopic procedure. Each stool specimen was examined using wet mount preparations following standard operating procedures to keep the quality of the result. A standard (not too thin and thick) fecal smear was prepared on the microscopic slide using 0.85% physiological saline. The smear was covered with a 22 by 22 mm cover slip. Then, the entire area was examined under a compound microscope using 10 and 40 times objectives [18]. Finally, the result was recorded in a reporting sheet prepared for data recording.

#### 2.4.3. Data Quality Control

The stool specimen was collected using a dry, clean, and leak-proof plastic stool-cup. The purity of stool collection cups and applicator sticks were checked visually. All smear preparations of the participants were checked by two laboratory personnel for confirmation of the result. If there was disagreement between two observers, the third laboratory personnel checked the result.

#### 2.4.4. Data Analysis and Interpretation

Data were entered and analyzed using SPSS version 20.0 (SPSS Inc., Chicago, 2011) software. Then, the study findings were explained in words, tables, and graphs. Univariate and multivariate logistic regression were used for statistical analysis to measure the significant association between the outcome and independent variables. *P* value < 0.05 was considered as statistically significant.

#### 2.4.5. Ethical Consideration

The study was carried out after ethical approval (Ref No.: HSC/R/C/Ser/Co/230/11/13) is obtained from the Research and Ethics Committee of Health Science College, Debre Markos University. Informed written consent was obtained from the study participants following an explanation of the objective of the study. For participants below the legal age, formal verbal consent was obtained from their parent/guardian. Stool examination was performed free of charge. Participants who were positive for intestinal parasite were linked to health center to have timely treatment.

#### 2.4.6. Operational Definitions

*Multigravida*: this refers to a woman who has been pregnant for at least a second time.

*Primigravida*: this refers to a woman who is pregnant for the first time.

## 3. Result

### 3.1. Characteristics of Study Participants

A total of 363 participants were included in this study, of which 56.2% (204/363) were in the age group 17-29 years old, and 73.3% (266/363) were farmers. The majorities (77.1%, 280/363) were multigravida, and 68.3% (248/363) were in the second trimester (Table 1).

### 3.2. Prevalence of Intestinal Parasites

The prevalence of at least one intestinal parasite was 43.5% (158/363) (95% CI: 38.3-48.2). Two species of protozoa and three species of helminths were identified. Of these five intestinal parasites identified, hookworm was the predominant (28.4%, 103/363) followed by *E. histolytica* (5.5%, 20/363) and *G. lamblia* (5.0%, 18/363). The ratio of protozoa to helminth infection was approximately 1 : 2.9. Of the total infections, 40.5% (147/363) were single infections and 3.0% (11/363) were mixed infections. All mixed infections were double infections. The highest case of double infection was noticed in hookworm and *E. histolytica/dispar* (5 cases) followed by hookworm and *Taenia* species (4 cases) (Table 2).

### 3.3. The Relative Proportion of Intestinal Parasites

**Figure 1:**
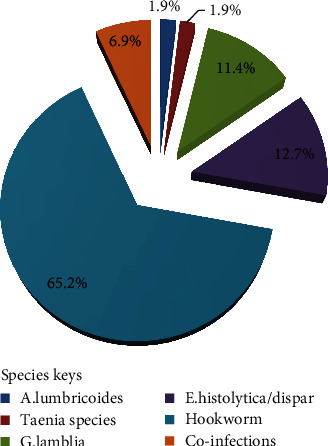
Relative proportion of intestinal parasites among pregnant women in Debre Elias District, March 2021-July 2021.

Among the positive cases identified, hookworm was the predominant (65.2%, 103/158) of all intestinal parasites identified followed by *E. histolytica/dispar* (12.7%, 20/158) and *G. lamblia* (11.4%, 18/158). *Ascaris lumbricoides* and *Taenia species* constituted the least percentage (1.9%, 3/158 each). Coinfections that are double constituted 6.9% (11/158) (Figure 1).

### 3.4. Factors Associated with Intestinal Parasitic Infection

Assessment of factors generally showed that occupation and source of drinking water were significantly associated with IPI (*P* < 0.05). Pregnant women who were farmers were 6.41 times (AOR = 6.41, 95% CI: 1.05-39.16) more likely to be infected by intestinal parasites than others, and pregnant women who were housewives were 8.84 times (AOR = 8.84, 95% CI: 1.29-60.28) more likely to be infected by intestinal parasites. Pregnant women who drank tape water were 0.52 times less (AOR = 0.52, 95% CI: 0.30-0.88) likely to be infected by intestinal parasites. No significant association was observed between IPI and educational status, frequency of shoe wear, trimester, and gravidity (*P* > 0.05) (Table 3).

## 4. Discussion

In Ethiopia, health care coverage to combat infectious diseases is minimal. Hence, the burden of intestinal parasitosis and other neglected parasitic diseases has been continued. Intestinal parasitosis continued to be the major public health concern causing substantial illness and death. Women of the child bearing age group are commonly affected by both protozoan and helminthic parasites. According to a recently published review report done by Chelkeba et al., the overall prevalence of IPI was 29%, hookworm being the predominant isolate followed by *A. lumbricoides* and *E. histolytica* [19]. Another most recent review also revealed the pooled prevalence of intestinal parasitic infection to be 27.3%. Hookworm and *Ascaris lumbricoides* have been the most prevalent parasite species [20]. These two reviews reported that Oromia and Amhara regions had the highest prevalence of intestinal parasites in pregnant women compared to others.

In the present study, the overall prevalence of at least one intestinal parasite was 43.5% as examined by the direct wet mount method. Five intestinal parasite species were identified; hookworm (28.4%), *E. histolytica/dispar* (5.5%), *G. lamblia* (5.0%), *Taenia species* (0.8%), and *A. lumbricoides* (0.8%). Mixed infection was noted accounting for 3.0% of parasitic infections. Among the positive cases, hookworm was found to be the predominant intestinal parasite identified (65.2%) followed by *E. histolytica/dispar* (12.7%) and *G. lamblia* (11.4%). *A. lumbricoides* and *Taenia species* accounted for the least percentage (1.9% each). This finding is in line with a study done in Hawassa, Southern Ethiopia, with an overall intestinal parasitic infection rate of 45.9%, *A. lumbricoides* being the predominant isolate followed by *S.mansoni* [21]. The present finding was also supported by a study conducted by Bolka and Gebremedhin (38.7%), *A. lumbricoides*, hookworm, *G. lamblia*, and *E. histolytica* being the predominant parasitic species isolated [22], and Abraham et a. (43.8%) with the predominance of hookworm and *A. lumbricoides* [23]. A study conducted in West Gojjam Zone, Northwest Ethiopia, also reported an overall intestinal parasite prevalence of 37.3%, hookworm being the leading cause of IPI followed by *E. histolytica/dispar* and *G. lamblia* [24].

In contrast, this finding is lower than a study done at Yifag Health Center, Northwest Ethiopia, with an intestinal parasite prevalence of 53.4% among pregnant women. *Taenia species*, *G. lamblia*, *E. histolytica*/*dispar*, hookworms, *A. lumbricoides, S. mansoni*, *H. nana*, *S. stercoralis*, and *E. vermicularis* were the identified intestinal parasites in the decreasing order of proportion [25]. As reported by Derso et al., the present study was higher than a study conducted in Felege Hiwot Referral Hospital (31.5%) with a predominance of *G. lamblia* followed by *E. histolytica* and hookworm [26]. The variation in prevalence of intestinal parasites might be due to differences in geographical location, climatic condition, hygienic practice, shoes wearing habits, and level of awareness.

Regression analysis of potential factors revealed that the source of drinking water and occupation (being a farmer) of pregnant women had statistically significant association with intestinal parasite infection (*P* < 0.05). The present finding was supported by Hailu et al. [24] and Yesuf et al. [23] who reported occupation (being farmer) as a significantly associated factor for IPIs. Eating raw vegetables and raw meat and having poor personal hygiene were also reported previously to be associated factors of intestinal parasitic infections [25].

### 4.1. Strength and Limitations of the Study

This study was a community-based study which can be representative and estimate the actual prevalence of intestinal parasites. However, use of only saline wet mount to detect intestinal parasites might underestimate the prevalence of intestinal parasites.

### 4.2. Conclusion and Recommendation

This study demonstrated that the prevalence of at least one intestinal parasite among pregnant women was 43.5%, and hookworm was found to be the predominant intestinal parasite followed by *E. histolytica/dispar* and *G. lamblia*. Prevalence of intestinal parasitic infection was significantly associated with occupation and source of drinking water. This magnitude highlights the need for control programs. Appropriate intervention measures targeting pregnant women should be implemented to prevent the burden inflicted by intestinal parasite infection and associated morbidities. Community-based intestinal parasite screening and treatment and improvement of environmental sanitation practice are highly recommended which can be achieved by the involvement of health extension workers and/or other health care providers, community, and religious leaders. Pregnant women should be encouraged to have uninterrupted antenatal care (ANC) visits and checked for any intestinal parasite regardless of signs and symptoms.

## Figures and Tables

**Table 1:** Sociodemographic profile of pregnant women at Debre Elias district, March 2021 to July 2021.

Variables	Category	Frequency (*n*)	Percentage (%)
Age group	17-29	204	56.2
	30-34	98	26.9
	35-39	55	15.2
	40 & above	6	1.7
Educational status	Unable to read and write	217	59.8
	Read and write	60	16.5
	Primary school	69	19
	Secondary school	17	4.7
Occupation	Farmer	266	73.3
	Merchant	59	16.2
	Housewife	32	8.8
	Government employee	6	1.7
Have shoes	Yes	347	95.6
	No	16	4.4
Source of water	Tape	175	48.2
	Spring	102	28.1
	Well	65	17.9
	River	21	5.8
Gravida	Primigravida	83	22.9
	Multigravida	280	77.1
Trimester	First	53	14.6
	Second	248	68.3
	Third	62	17.1

**Table 2:** Prevalence of intestinal parasites among pregnant women in Debre Elias district, Northwest Ethiopia, March 2021 to July 2021.

Parasite species		Frequency (*N*)	Percentage (%)
Monoinfection	*Entamoeba histolytica/dispar*	20	5.5%
	*Giardia lamblia*	18	5.0%
	Hookworm	103	28.4%
	*Ascaris lumbricoides*	3	0.80%
	*Taenia species*	3	0.80%
	Total	147	40.5%
Double infection	Hookworm+*Taenia species*	4	1.1%
	Hookworm+*E. histolytica/dispar*	5	1.4%
	Hookworm+*Giardia lamblia*	2	0.5%
	Total	11	3.0%
Overall total		158	43.5%

N.B: both single and mixed infections were considered to calculate the prevalence.

**Table 3:** Bivariate and multivariate analysis of factors associated with intestinal parasites among pregnant women at Debre Elias district, March 2021 to July 2021.

Variables	Category	Result				*P* value
		Positive	Negative	COR (95% CI)	AOR (95% CI)	
Educational status	Unable to read & write	112	105	0.52 (0.18-1.43)	0.37 (0.12-1.13)	0.08
	Read & write	19	41	1.18 (0.38-3.66)	1.04 (0.32-3.39)	0.95
	Primary school	21	48	1.25 (0.41-3.82)	0.86 (0.26-2.83)	0.81
	Secondary school	6	11	1.00	1.00	
Occupation	Farmer	119	147	2.47 (0.45-13.72)	6.41 (1.05-39.16)	0.034^∗^
	Merchant	26	33	2.54 (0.43-14.95)	4.62 (0.73-29.26)	0.104
	Housewife	9	23	5.11 (0.79-32.97)	8.84 (1.29-60.28)	0.026^∗^
	Government employ	4	2	1.00	1.00	
Source of water	Tape	64	111	0.55 (0.34-0.91)	0.52 (0.30-0.88)	0.017^∗^
	Spring	52	50	1.05 (0.41-2.70)	0.77 (0.29-2.05)	0.60
	River	11	10	1.20 (0.45-3.23)	0.80 (0.28-2.28)	0.68
	Well	31	34	1.00	1.00	
Shoe wearing habit	Yes	151	196	1.00	1.00	
	No	7	9	1.01 (0.37-2.77)		
Frequency of shoe use	Always	81	133	1.00	1.00	
	Sometimes	68	60	0.54 (0.34-0.84)	0.70 (0.42-1.19)	0.19
	Never	2	3	0.91 (0.15-5.58)	0.78 (0.12-5.04)	0.79
Gravida	Primigravida	32	51	1.00	1.00	
	Multigravida	126	154	0.77 (0.46-1.26)	0.99 (0.50-1.98)	0.99
Trimester	First	25	28	0.81 (0.39-1.69)	0.72 (0.32-1.59)	0.41
	Second	107	141	0.95 (0.54-1.67)	1.05 (0.57-1.93)	0.88
	Third	26	36	1.00	1.00	

^∗^Statistically significant; AOR: adjusted odds ratio; COR: crude odds ratio; CI: confidence interval.

## Data Availability

The data sets used and/or analyzed during the current study are available from the corresponding author upon reasonable request.

## Abbreviations

IPI: Intestinal parasite infection
WHO: World Health Organization.
